# Supplementary material for: OpenMM 7: Rapid development of high performance algorithms for molecular dynamics
Source: PLoS Comput Biol. 2017 Jul 26;13(7):e1005659. doi: 10.1371/journal.pcbi.1005659 (PMC5549999; doi:10.1371/journal.pcbi.1005659)
Supplement: S1 Source Code — (ZIP) [file pcbi.1005659.s002.zip › OpenMM-7.0.1-Source/docs/api-c++/generated/OpenMM.RBTorsionForce.html]

RBTorsionForce — OpenMM @OPENMM\_MAJOR\_VERSION@.@OPENMM\_MINOR\_VERSION@ documentation


# RBTorsionForce¶

*class* `OpenMM::``RBTorsionForce`¶
:   This class implements an interaction between groups of four particles that varies with the torsion angle between them according to the Ryckaert-Bellemans potential. To use it, create an `RBTorsionForce` object then call `addTorsion()` once for each torsion. After a torsion has been added, you can modify its force field parameters by calling `setTorsionParameters()`. This will have no effect on Contexts that already exist unless you call `updateParametersInContext()`.

    Methods

    |  |  |
    | --- | --- |
    | `RBTorsionForce` | Create a `RBTorsionForce`. |
    | `getNumTorsions` | Get the number of Ryckaert-Bellemans torsion terms in the potential function |
    | `addTorsion` | Add a Ryckaert-Bellemans torsion term to the force field. |
    | `getTorsionParameters` | Get the force field parameters for a Ryckaert-Bellemans torsion term. |
    | `setTorsionParameters` | Set the force field parameters for a Ryckaert-Bellemans torsion term. |
    | `updateParametersInContext` | Update the per-torsion parameters in a `Context` to match those stored in this `Force` object. |
    | `usesPeriodicBoundaryConditions` | Returns whether or not this force makes use of periodic boundary conditions. |

    `RBTorsionForce`()¶
    :   Create a `RBTorsionForce`.

    int `getNumTorsions`() *const*¶
    :   Get the number of Ryckaert-Bellemans torsion terms in the potential function

    int `addTorsion`(int *particle1*, int *particle2*, int *particle3*, int *particle4*, double *c0*, double *c1*, double *c2*, double *c3*, double *c4*, double *c5*)¶
    :   Add a Ryckaert-Bellemans torsion term to the force field.

        |  |  |
        | --- | --- |
        | Parameters: |  |

        - **particle1** – the index of the first particle forming the torsion
        - **particle2** – the index of the second particle forming the torsion
        - **particle3** – the index of the third particle forming the torsion
        - **particle4** – the index of the fourth particle forming the torsion
        - **c0** – the coefficient of the constant term, measured in kJ/mol
        - **c1** – the coefficient of the 1st order term, measured in kJ/mol
        - **c2** – the coefficient of the 2nd order term, measured in kJ/mol
        - **c3** – the coefficient of the 3rd order term, measured in kJ/mol
        - **c4** – the coefficient of the 4th order term, measured in kJ/mol
        - **c5** – the coefficient of the 5th order term, measured in kJ/mol

        |  |  |
        | --- | --- |
        | Returns: | the index of the torsion that was added |

    void `getTorsionParameters`(int *index*, int &*particle1*, int &*particle2*, int &*particle3*, int &*particle4*, double &*c0*, double &*c1*, double &*c2*, double &*c3*, double &*c4*, double &*c5*) *const*¶
    :   Get the force field parameters for a Ryckaert-Bellemans torsion term.

        |  |  |
        | --- | --- |
        | Parameters: |  |

        - **index** – the index of the torsion for which to get parameters
        - **particle1** – [out] the index of the first particle forming the torsion
        - **particle2** – [out] the index of the second particle forming the torsion
        - **particle3** – [out] the index of the third particle forming the torsion
        - **particle4** – [out] the index of the fourth particle forming the torsion
        - **c0** – [out] the coefficient of the constant term, measured in kJ/mol
        - **c1** – [out] the coefficient of the 1st order term, measured in kJ/mol
        - **c2** – [out] the coefficient of the 2nd order term, measured in kJ/mol
        - **c3** – [out] the coefficient of the 3rd order term, measured in kJ/mol
        - **c4** – [out] the coefficient of the 4th order term, measured in kJ/mol
        - **c5** – [out] the coefficient of the 5th order term, measured in kJ/mol

    void `setTorsionParameters`(int *index*, int *particle1*, int *particle2*, int *particle3*, int *particle4*, double *c0*, double *c1*, double *c2*, double *c3*, double *c4*, double *c5*)¶
    :   Set the force field parameters for a Ryckaert-Bellemans torsion term.

        |  |  |
        | --- | --- |
        | Parameters: |  |

        - **index** – the index of the torsion for which to set parameters
        - **particle1** – the index of the first particle forming the torsion
        - **particle2** – the index of the second particle forming the torsion
        - **particle3** – the index of the third particle forming the torsion
        - **particle4** – the index of the fourth particle forming the torsion
        - **c0** – the coefficient of the constant term, measured in kJ/mol
        - **c1** – the coefficient of the 1st order term, measured in kJ/mol
        - **c2** – the coefficient of the 2nd order term, measured in kJ/mol
        - **c3** – the coefficient of the 3rd order term, measured in kJ/mol
        - **c4** – the coefficient of the 4th order term, measured in kJ/mol
        - **c5** – the coefficient of the 5th order term, measured in kJ/mol

    void `updateParametersInContext`(Context &*context*)¶
    :   Update the per-torsion parameters in a `Context` to match those stored in this `Force` object. This method provides an efficient method to update certain parameters in an existing `Context` without needing to reinitialize it. Simply call `setTorsionParameters()` to modify this object’s parameters, then call `updateParametersInContext()` to copy them over to the `Context`.

        The only information this method updates is the values of per-torsion parameters. The set of particles involved in a torsion cannot be changed, nor can new torsions be added.

    bool `usesPeriodicBoundaryConditions`() *const*¶
    :   Returns whether or not this force makes use of periodic boundary conditions.

        |  |  |
        | --- | --- |
        | Returns: | true if force uses PBC and false otherwise |

High performance molecular simulation on GPUs


### Navigation

- Core Objects
- Forces
- Integrators
- Extras

©2015, Stanford University and the Authors.
|
Powered by Sphinx 1.3.1
& Alabaster 0.7.7
|
Page source
